# Myoblast‐derived exosomes promote the repair and regeneration of injured skeletal muscle in mice

**DOI:** 10.1002/2211-5463.13504

**Published:** 2022-11-11

**Authors:** Shusen Ji, Pei Ma, Xiaorui Cao, Juan Wang, Xiuju Yu, Xiaomao Luo, Jiayin Lu, Wei Hou, Zhijuan Zhang, Yi Yan, Yanjun Dong, Haidong Wang

**Affiliations:** ^1^ College of Veterinary Medicine Shanxi Agricultural University Jinzhong China; ^2^ Department of Nephrology, Shanghai General Hospital Shanghai Jiao Tong University School of Medicine China; ^3^ Fenyang Hospital of Shanxi Province China; ^4^ College of Veterinary Medicine China Agricultural University Beijing China

**Keywords:** C2C12‐derived exosomes, fibro‐adipogenic progenitors, myoblast, repair and regeneration, satellite cells, skeletal muscle

## Abstract

When skeletal muscle is damaged, satellite cells (SCs) are activated to proliferate rapidly and fuse with the damaged muscle fibers to form new muscle fibers, thereby promoting muscle growth and remodeling and repair of trauma. Exosomes from differentiating human skeletal muscle cells trigger myogenesis of stem cells and provide biochemical cues for skeletal muscle regeneration. Therefore, we hypothesized that, when muscles are injured, myoblast‐derived exosomes may regulate muscle repair and regeneration. Here, we investigated the underlying mechanism by applying C2C12‐derived exosomes to injured mouse skeletal muscles. The expression levels of skeletal muscle regeneration factors paired box 7 and lipid‐promoting factor peroxisome proliferator‐activated receptor γ were upregulated, whereas the expression levels of fibrosis factors collagen‐1 and α‐smooth muscle actin decreased. The expression of proliferating cell nuclear antigen was elevated after applying C2C12‐derived exosomes to SCs. Application of C2C12‐derived exosomes to fibro‐adipogenic progenitors resulted in an increase in peroxisome proliferator‐activated receptor γ expression and adipogenesis capacity, whereas α‐smooth muscle actin expression and fibrosis capacity decreased. Analysis of the transcriptome and proteome of SCs after treatment with exosomes showed the involvement of multiple biological processes, including proliferation and differentiation of SCs, muscle regeneration, skeletal muscle atrophy, and the inflammatory response after muscle injury. Hence, our data suggest that C2C12‐derived exosomes can promote the regeneration of skeletal muscle fibers, accelerate the production of fat from damaged muscles, inhibit the fibrosis of damaged muscles, and accelerate injury repair, which is related to exosome‐mediated regulation of the proliferation of SCs, differentiation of fibro‐adipogenic progenitors, and modulation of SC mRNA expression and protein formation and decomposition.

## Graphical abstract

In the present study, C2C12‐derived exosomes were applied to injured mouse skeletal muscle, satellite cells (SCs) and fibro‐adipogenic progenitors (FAPs) and it was found that they could promote the regeneration of skeletal muscle fibers, accelerate the proliferation of SCs, promote the differentiation of FAPs into adipocytes and inhibit fibrosis, thus accelerating the repair of injured muscle.

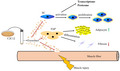

**Abbreviations**
α‐SMA: α‐smooth muscle actin
CD9: CD9 molecule
EB: Evans blue
FAPs: fibro‐adipogenic progenitors
GAPDH: Glyceraldehyde‐3‐phosphate dehydrogenase
GAS: gastrocnemius
HE: hemotoxylin and eosin
IL: interleukin
MAPK: mitogen‐activated protein kinase
Myf5: myogenic factor 5
MyoD: myogenic differentiation antigen
NF‐κB: nuclear factor kappa B
NTA: nanoparticle tracking analysis
Pax7: paired box 7
PCNA: proliferating cell nuclear antigen
PPARγ: peroxisome proliferator‐activated receptor γ
SCs: satellite cells
TEM: transmission electron microscope
TNF: tumor necrosis factor
TSG101: tumor susceptibility gene 101
α‐SMA: α‐smooth muscle actin

Skeletal muscle is the largest organ in animal organisms. After acute or chronic injury, skeletal muscles promote the repair of injuries through complex interactions between primitive muscles and circulating cell populations [1, 2, 3, 4]. Myogenic progenitor cells proliferate and differentiate into myoblasts under the action of some factors [paired box 7 (Pax7), myogenic differentiation antigen (MyoD), myogenic factor 5, etc.] and myoblasts fuse to form multinucleated skeletal muscle fibers. Satellite cells (SCs) are the main stem cells directly responsible for forming new fibers in skeletal muscle [5, 6, 7]. When skeletal muscle is damaged, SCs are activated to proliferate rapidly and fuse with the damaged muscle fibers to form new muscle fibers, thereby promoting muscle growth and remodeling and repair of trauma [8, 9, 10]. Fibro‐adipogenic progenitors (FAPs) comprise mesenchymal‐derived muscle mesenchymal stem cell populations [11], which can differentiate into intramuscular fat cells or fibroblasts that play an active role in muscle injury repair and regeneration.

At present, exosomes are an important medium of cell communication; they express tumor susceptibility gene 101 (TSG101) and CD9 molecule (CD9) proteins, and can exchange various proteins, lipids and mRNA with peripheral or distal cells [12, 13, 14]. Previous studies reported that cancer cell exosomes have the regulatory function of tissue damage repair [15]. Exosomes from differentiating human skeletal muscle cells trigger myogenesis of stem cells and provide biochemical cues for skeletal muscle regeneration [16]. Stem cell‐derived exosomes prevent pyroptosis and repair ischemic muscle injury [17]. Myotube‐exosome microRNAs could contribute to the commitment of myoblasts in the process of differentiation [18].

Therefore, we speculate that, when muscles are injured, myoblast‐derived exosomes can regulate the proliferation of SCs and the differentiation of FAPs, regulate muscle repair and regeneration, and then affect animal muscle development. The present study aims to reveal the exosome‐mediated interaction between muscle tissue cells and lay a solid theoretical foundation for the use of exosomes to treat muscle injuries.

## Materials and methods

### C2C12‐derived exosome identification

When C2C12 was differentiated to 80%, serum‐free Dulbecco's modified Eagle's medium (BI, Kibbutz Beit‐Haemek, Israel) was added for culture for 24–36 h. The supernatant was collected and the exosomes were isolated by ultra‐high‐speed centrifugation (Preparative Ultracentrifuges, Hitachi, Japan). The gravity of the centrifugation and time were in order: 300 ***g***, 10 min; 2000 ***g***, 10 min; 10 000 ***g***, 30 min; 100 000 ***g***, 90 min; and 100 000 ***g***, 90 min. Then, nanoparticle tracking analysis (NTA), transmission electron microscope (TEM) detection, and western blotting identification of specific proteins were performed.

### Animals

Thirty‐five 6‐week‐old male C57BL/6 mice (Vital River, Beijing, China) were selected and divided into blank control group, injury group, and exosome treatment group [Injury: 1.2% BaCl_2_ was injected into the gastrocnemius (GAS) of mice; Treatment: 6.4 × 10^10^ pieces·mL^−1^, 50 μL of exosomes were used for treatment every 2 days]. The intact GAS was removed for a follow‐up trial.

### Ethical approval

All animal care and experimental protocols complied with the Animal Management Rule of the Ministry of Health, People's Republic of China (Documentation No. 55, 2001) and the Guide for the Care and Use of Laboratory Animals published by the United States National Institutes of Health (Publication No. 85‐23, Revised 1996), as well as the Global Research Animal Guide. All animal operations were carried out in accordance with the ‘Guidelines for the Care and Use of Laboratory Animals of Shanxi Agricultural University’ and were approved by the Animal Medicine Committee of Shanxi Agricultural University [SXAU‐EAW‐2020M0725].

### Hemotoxylin and eosin (HE) and Evans blue (EB) staining

The GAS was submerged with OCT embedding agent (Solarbio, Beijing, China). After it was frozen, a tissue section with a thickness of 5 μm was made with a frozen slicer (Leica, Wetzlar, Germany). The tissue sections were stained with HE.

At 16–24 h before tissue sampling, 1% volume of Evans blue dye solution was injected intraperitoneally, and the fresh muscles showed a distinct ‘blue’ with the naked eye after inspection. The size of the blue area was used to determine the degree of skeletal muscle damage.

### Flow sorting of SCs and FAPs

The limb muscles of mice were taken, chopped, and digested at 37 °C with collagenase 2 (800 U·mL^−1^; Gibco, Waltham, MA, USA) for 30 min, then digested with Collagenase D and Dispase II (Roche, Basel, Switzerland) for 1 h. The resulting suspension was filtered with a 45‐μm cell strainer. Filter of FAPs and SCs was conducted by CD31^−^/CD45^−^/ Sca‐1^+^/Integrin‐α7^+^ (553372/553080/553336/FAB3518A; BD Pharmingen, San Diego, CA, USA) scheme in Flow cytometer (BD FACS Aria III, USA; BD Biosciences, Franklin Lakes, NJ, USA). In the forward scatter/side scatter plot, the living cell group was determined to set P1 gate, the apoptosis cells and cell fragments were excluded, and the P2 gate was determined according to the target cells; P3 gate is CD31^−^/CD45^−^ (FITC) negative cell group, P4 gate is Sca‐1^+^ (PE‐Texas Red‐A/PE‐CF594) positive marker FAPs, and P5 gate is Integrin‐α7^+^ (APC) positive marker SCs.

### Cellular immunofluorescence

After cells climbing slides, the sample was fixed with 4% cold paraformaldehyde for 30 min and 0.1% Triton‐100 for 10 min to break the cell membrane. Then, 5% goat serum solution was added to block for 30 min. Immunofluorescence staining was performed with anti‐Pax7 (20570‐1‐AP; Proteintech, Wuhan, China) and MyoD (ab16148; Abcam, Cambridge, UK) antibodies. Secondary antibodies, anti‐rat (CW0102S; CWBIO, Taizhou, China) and anti‐rabbit (CW0103S; CWBIO), were added at 37 °C for 1 h. The sample was sealed with an anti‐fluorescence decay seal containing 4′,6‐diamidino‐2‐phenylindole (Solarbio) and observed under fluorescence microscope (Nikon, Tokyo, Japan).

### Assessment of cell viability by CCK‐8

Approximately 100 μL of the SCs suspension was configured in a 96‐well plate. After preculture for 24 h, 10 μL of C2C12‐derived exosomes was added at different concentrations. The common medium was added to the control group. Then, 10 μL of CCK‐8 solution (Yeasen, Shanghai, China) was added to each well followed by incubation for 2 h. Absorbance was determined at 450 nm with a microplate reader.

### Western in cell

The SCs were uniformly inoculated in a black background 96‐well plate and then exosomes were added when it grew to 50–60%. The sample was fixed with 4% paraformaldehyde for 20 min. After 24–32 h, the sample 3% Triton was added for 20 min, subjected to 5% BSA blocking for 1 h, and then primary antibody was added for incubation overnight at 4 °C. The primary antibody was recovered the next day and secondary antibody was added in the dark place at room temperature for 1 h. After thorough drying, the sample was imaged in a NIR Near‐Infrared Laser Scanning Imaging System (Azure, Dublin, CA, USA).

### Real‐time fluorescent quantitative PCR and western blotting

cDNA was synthesized in accordance with the instructions provided in the reverse transcription kit (Vazyme, Shanghai, China). SYBR, diethyl pyrocarbonate water, cDNA, β‐actin as the reference gene, and upstream and downstream primers were mixed proportionally to 10 μL for the polymerase chain reaction. The primer sequences are shown in Table S3.

After the concentration was determined by the BCA method, SDS/PAGE electrophoresis was performed and the target protein was transferred to the nitrocellulose membrane. The membrane was incubated with primary antibody overnight at 4 °C, followed by secondary antibody for 1 h at 37 °C. The protein was visualized with a gel imager (Bio‐Rad, Hercules, CA, USA). The primary antibodies included Pax7 and anti‐MyoD (ab199010 and ab16148; Abcam); PPARγ and Glyceraldehyde 3‐phosphate dehydrogenase (16643‐1‐AP and 60004‐1‐AP; Proteintech); α‐smooth muscle actin (α‐SMA; 19245, Cell Signaling, Danvers, MA, USA); Collagen‐1 (ab255809; Abcam); anti‐β‐actin (CW0096M; CWBIO), and TSG101 and CD9 (28283‐1‐AP and 20597‐1‐AP; Proteintech).

### Transcriptome and proteome sequencing

Sample (SCs and SCs‐exo) RNAs were extracted to detect their purity, concentration, and integrity. A library was constructed after qualification, and the effective concentration of the library (the effective concentration of the library > 2 nm) was accurately quantified by a quantitative PCR method. After the library inspection was qualified, different libraries were pooled according to the target data volume and sequenced with the Illumina HiSeq 2500 (BioMarker, Beijing, China) platform.

Enzymolysis was performed after the extraction of the sample protein, and enzymolysis efficiency was determined with a Q Extractive HF spectrometer (Thermo Fisher, Waltham, MA, USA). The samples were resuspended in an aqueous solution containing 5% formic acid. The polypeptides were isolated by the UPLC M‐Class system (Waters Corp., Milford, MA, USA) in the autosampler and then sprayed into the mass spectrometer. C18 RP analysis column (particle size 1.8 μm, inner diameter 100 μm × 250 mm; Waters Corp.) was used for liquid phase separation. A linear gradient was used to set the flow rate to 300 nL·min^−1^ for the entire elution process. Finally, label‐free quantitative analysis was carried out based on the results of the raw file using proteome discoverer (Thermo Fisher).

### Statistical analysis

image lab (Bio‐Rad) was used to analyze the results of the western blot analysis. The relative expression level of the target protein was calculated from the ratio of the target to the internal reference. The quantitative PCR results were calculated based on the ΔΔCT value. Data processing was performed with one‐way analysis of variance using prism, version 5.0 (GraphPad Software Inc., San Diego, CA, USA).

## Results

### C2C12‐derived exosome identification

**Fig. 1:**
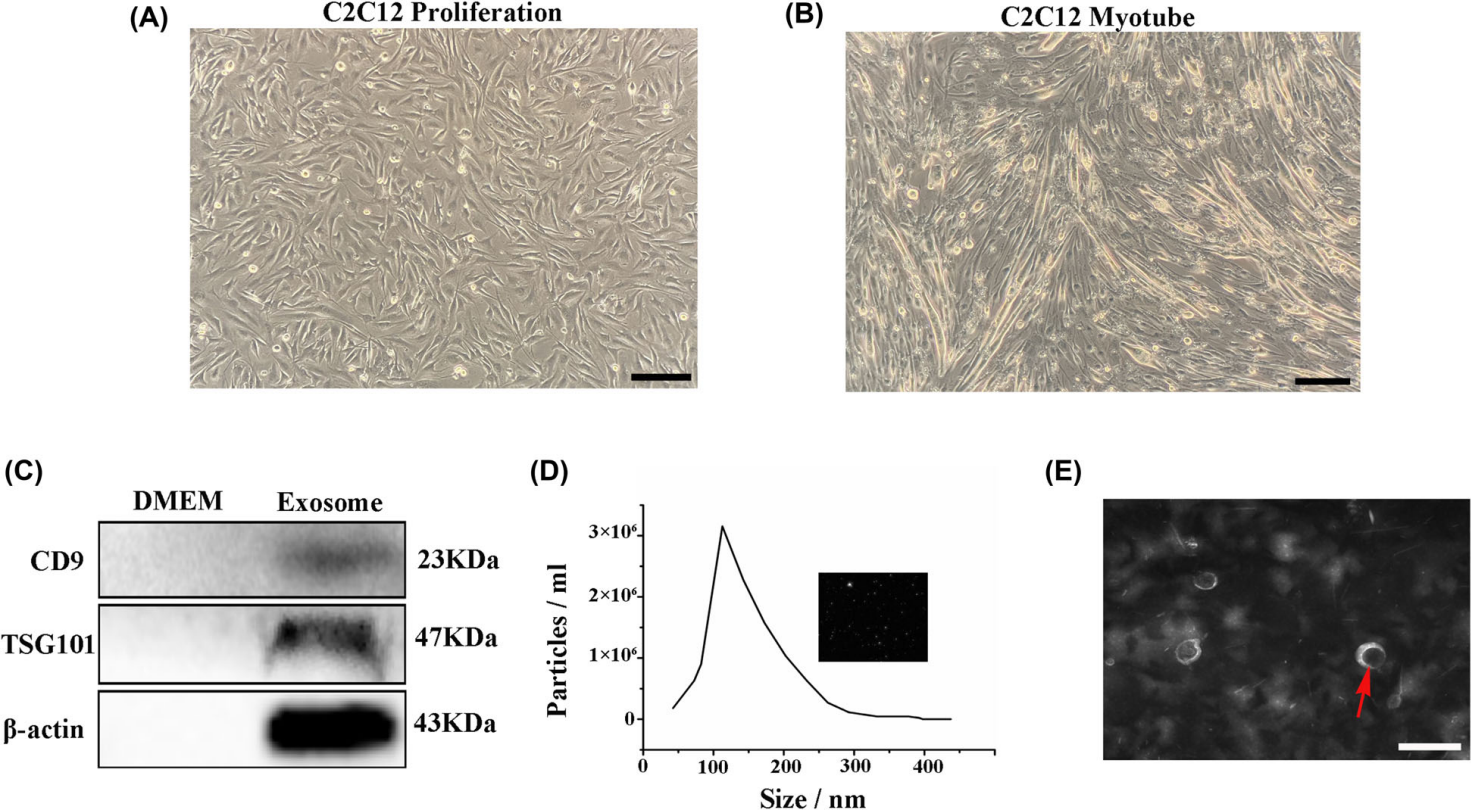
Characteristics of C2C12‐derived exosomes. (A) Undifferentiated morphology of C2C12 cells (10×). (B) C2C12 cells differentiate into myotube morphology (10×). (C) Western blotting identified the positive expression of exosome‐specific membrane proteins CD9 and TSG101. (D) NTA detection of exosome particle size, concentration, and exosome; screenshot of Brownian motion video. (E) TEM photographs showing that the exosomes have a bilayer membrane spherical vesicle structure, with a scale of 200 nm.

Exosomes were extracted after C2C12 differentiated into myotubes (Fig. 1A,B). Exosome‐tagged proteins TSG101 and CD9 were positive (Fig. 1C). NTA showed that the diameter of the C2C12‐derived exosomes was approximately 139.1 nm; the concentration was 6.4 E+10 particles·mL^−1^ (Fig. 1D). The brownian motion speed and the light intensity fluctuation was fast (Fig. 1D, upper right). The TEM image presented the structure of exosome as double‐membrane spherical vesicles (Fig. 1E).

### Effects of C2C12‐derived exosomes on the repair and regeneration of injured muscle

**Fig. 2:**
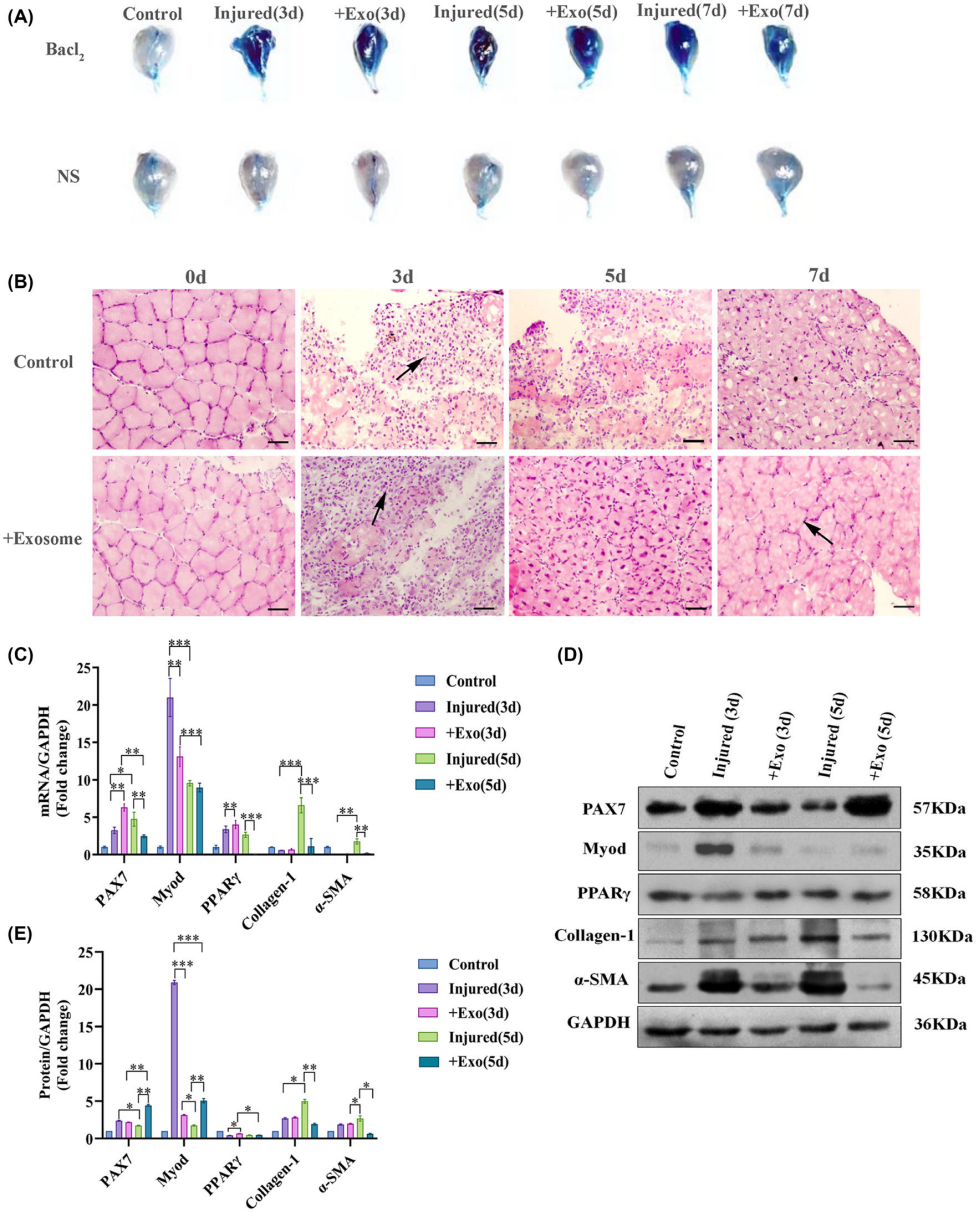
Histological evaluation of muscles and the expression of genes and proteins related to muscle regeneration. (A) According to the damage to the EB stained muscle fibers, the 5‐day C2C12‐derived exosomes treatment group has significantly less EB intake than the injury group (i, injury; e, exosome therapy; the same below). (B) HE staining results in the injury group and the exosome treatment group on days 3, 5, and 7. A large number of central nuclei appeared in the exosome treatment group on day 5, and repair was significantly faster than that in the injury group, whereas a large number of intact muscle fibers were seen in the treatment group on day 7, indicating that the treatment group regenerated faster than the injury group, with a scale of 200 μm. (C–E) The relative expression levels of Pax7, MyoD, PPARγ, collagen‐1, and α‐SMA proteins and mRNAs after C2C12‐derived exosomes to the GAS (*n* = 3 per group). The error bars indicate the SEM, whereas comparisons between two groups were performed by an unpaired Student's test, *P < 0.05; **P < 0.01; ***P < 0.001. Con, Control group; injured (3d), 3‐day injuried group; +Exo(3d), 3‐day C2C12‐derived exosomes treatment group; injured (5d), 5‐day injury group; +Exo(5d), 5‐day C2C12‐derived exosomes treatment group).

The EB staining showed that EB accumulation was greatest on day 3 of injury. As the time of injury increased, the degree of damage to the muscle fibers became larger and reached a maximum on day 5, and some erosion of the GAS was visible. On day 7, the EB uptake was significantly reduced, indicating that damaged muscle fibers began to repair. No difference on EB accumulation was detected between the C2C12‐derived exosomes treatment group and injury groups on day 3. However, from day 5 onwards, the treatment group significantly took up less EB (Fig. 2A).

Compared with the healthy control group, the HE staining revealed that the fibers in the damaged area of the injury group were polygonal in shape; the interstitial space between the fibers was enlarged and an obvious inflammatory phenomenon existed. In the injury groups, the damage to the muscle fibers was more severe on days 3 and 5, whereas, on day 7, the level of inflammation in the damaged muscles decreased, the central nucleus increased and the repair phase started. The exosome treatment group still had a large amount of inflammatory cell infiltration near the damaged area on day 3; the inflammatory invasion area was significantly reduced, and central nuclear muscle fibers appeared, showing a repair trend on day 5; intact muscle fibers were already visible on day 7 (Fig. 2B).

The experiment further detected the expression of muscle regeneration indicators. C2C12‐derived exosomes effectively promoted the increase in the expression of SCs marker Pax7. On day 5, the Pax7 protein expression of the exosome treatment group peaked, whereas Myod expression showed a trend of first decreasing and then increasing.

The effect of C2C12‐derived exosomes on the adipogenesis was further detected. On day 3, it appeared that the peroxisome proliferator‐activated receptor γ (PPARγ) protein expression increased 1.58‐fold times in the exosome group compared to that in the injury group; the exosome group increased 1.03‐fold times on day 5. There was an increase in the PPARγ mRNA expression in the exosome group on day 3 and a decrease in the PPARγ mRNA levels on day 5.

The effect of C2C12‐derived exosomes on skeletal muscle fibrosis were also evaluated. On day 3, the α‐SMA protein expression increased 1.05‐fold times in the exosome group but decreased significantly 0.24‐fold times in the exosome group on day 5. Collagen‐1 protein expression increased 1.04‐fold times in the exosome group on day 3 and decreased 0.39‐fold times in the exosome group on day 5. On day 3, Collagen‐1 and α‐SMA gene expression was higher in the exosome group; on day 5, it was lower in the exosome group compared to that in the injury group (Fig. 2C–E).

### Effects of C2C12‐derived exosomes on SCs appreciation and FAPs differentiation

**Fig. 3:**
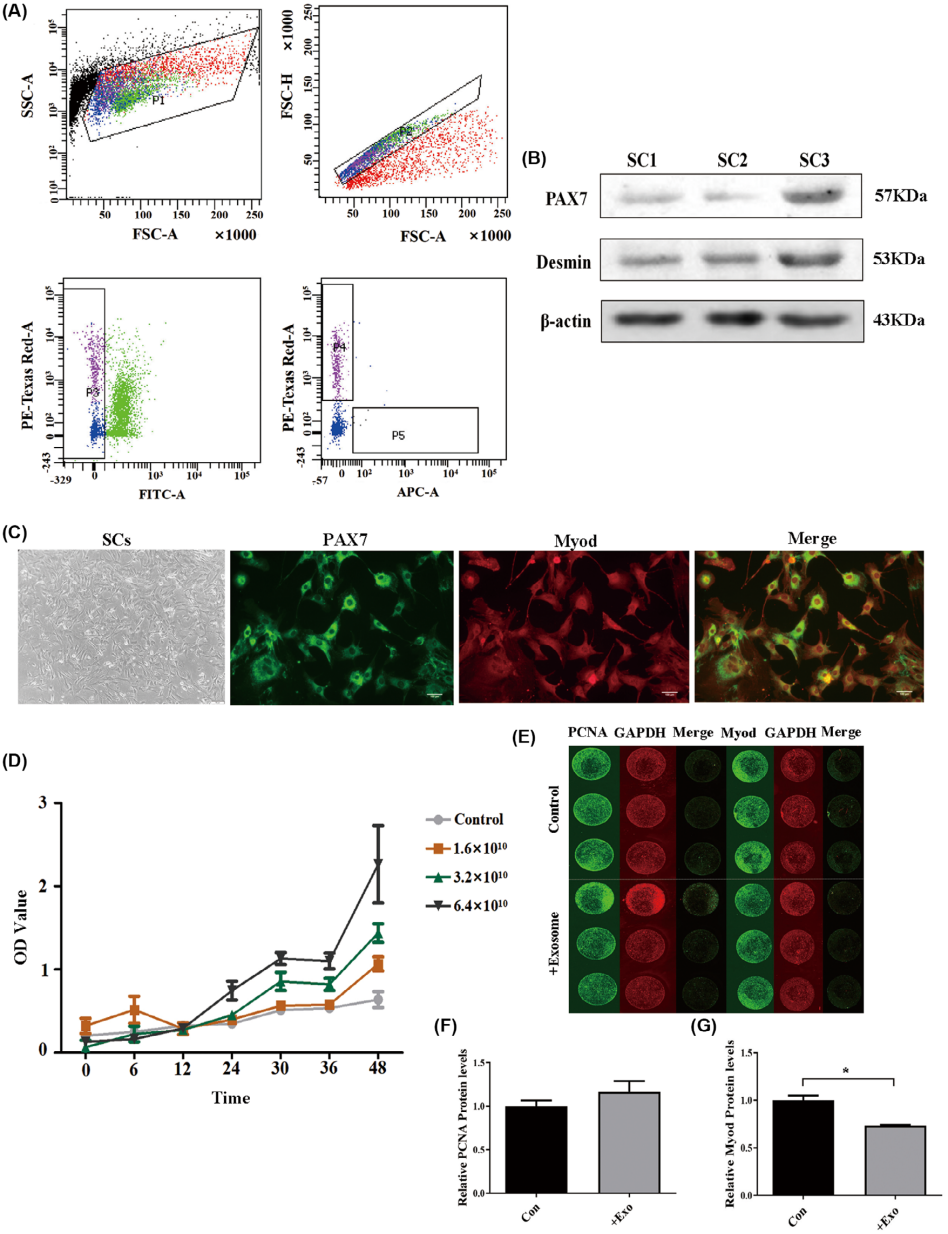
Effects of C2C12‐derived exosomes on the proliferation of SCs. (A) FAPs and SCs were filtered by CD31^−^/CD45^−^/Sca‐1^+^/integrin‐α7^+^, with P4 gate as FAPs and P5 gate as SCs. (B) Western blotting identified that the expressions of specific proteins Pax7 and Desmin of SCs were positive. (C) Immunofluorescence identified that SCs‐specific proteins Pax7 and Myod showed positive expression, with a scale of 100 μm. (D) Proliferative activity of SCs after C2C12‐derived exosome treatment (different colors in the upper right corner represent exosome concentration). (E–G) Western in cell detected the expression levels of PCNA and Myod protein of SCs after C2C12‐derived exosome treatment (*n* = 3 per group). The error bars indicate the SEM, whereas comparisons between two groups were performed by an unpaired Student's test, *P < 0.05; +exo, C2C12‐derived exosome treatment of SCs).

SCs and FAPs were obtained by flow sorting (Fig. 3A). We confirmed that Pax7 and Desmin were positive (Fig. 3B). Based on immunofluorescence staining, the cells showed red fluorescence (Pax7) and green light (MyoD) (Fig. 3C). Over time, the higher the concentration of C2C12‐derived exosomes, the stronger the proliferation activity (Fig. 3D). The exosome group had a slight increase in the proliferating cell nuclear antigen (PCNA) protein expression compared to that in the control group, and the expression level of the Myod protein decreased significantly (Fig. 3E–G).

**Fig. 4:**
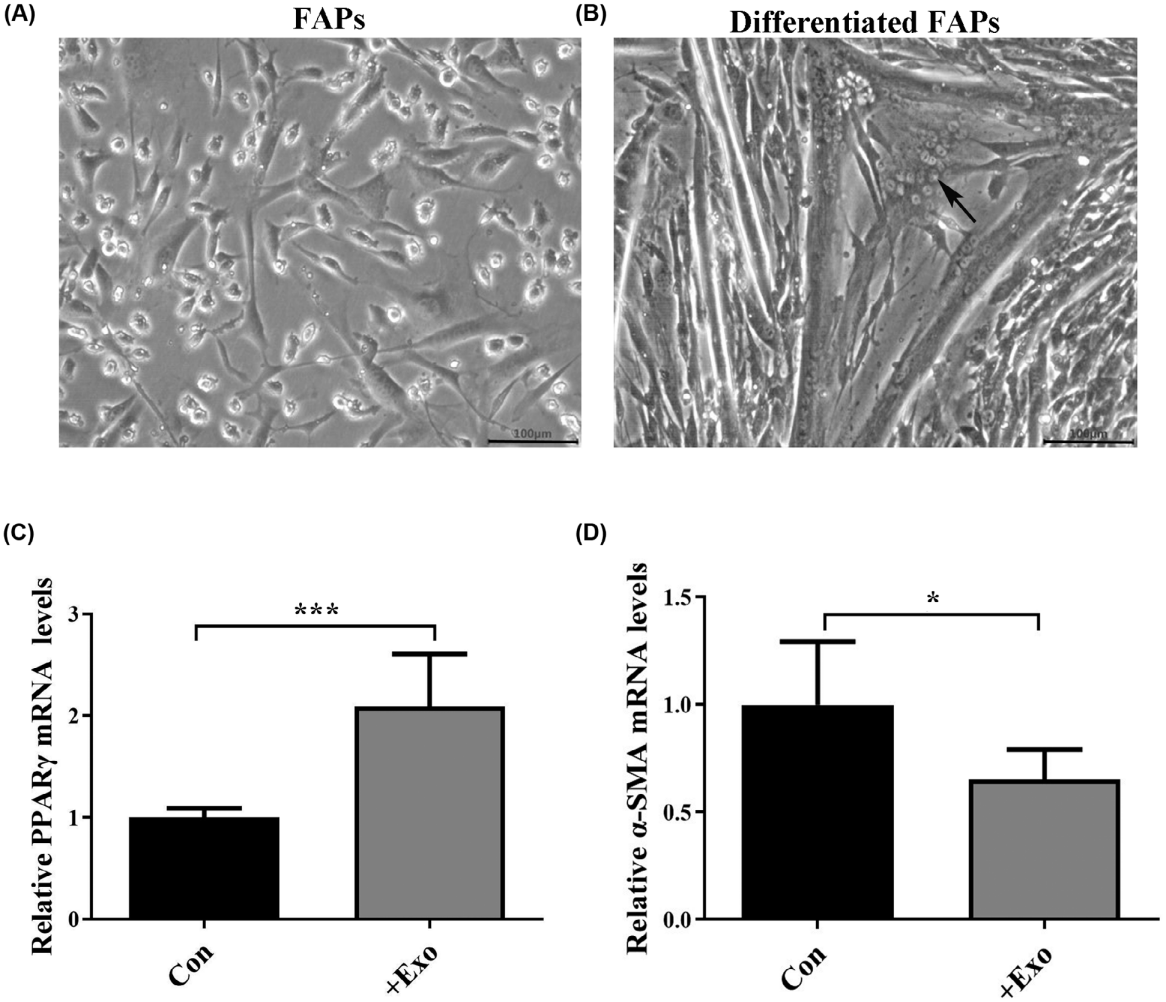
Effects of C2C12‐derived exosomes on FAPs differentiation. (A) Normal morphology of FAPs, with a scale of 100 μm. (B) Differentiation morphology of FAPs after treatment with C2C12‐derived exosomes, with a scale of 100 μm (the arrow points to lipid droplets). (C) Relative expression levels of FAPs α‐SMA mRNA after C2C12‐derived exosome treatment. (D) Relative expression levels of FAPs PPARγ mRNA after C2C12‐derived exosome treatment (*n* = 3 per group). The error bars indictae the SEM, whereas comparisons between two groups were performed by an unpaired Student's test, *P < 0.05; ***P < 0.001; +exo, C2C12‐derived exosome treatment of FAPs.

The addition of C2C12‐derived exosomes can promote FAP (Fig. 4A,B) differentiation. We detected that the expression of α‐SMA in the exosome group reduced 0.65‐fold times and the expression of PPARγ increased 2.09‐fold times (Fig. 4C,D).

### Analysis of the effect of C2C12‐derived exosomes on promoting the proliferation of SCs and muscle damage repair mechanism

**Fig. 5:**
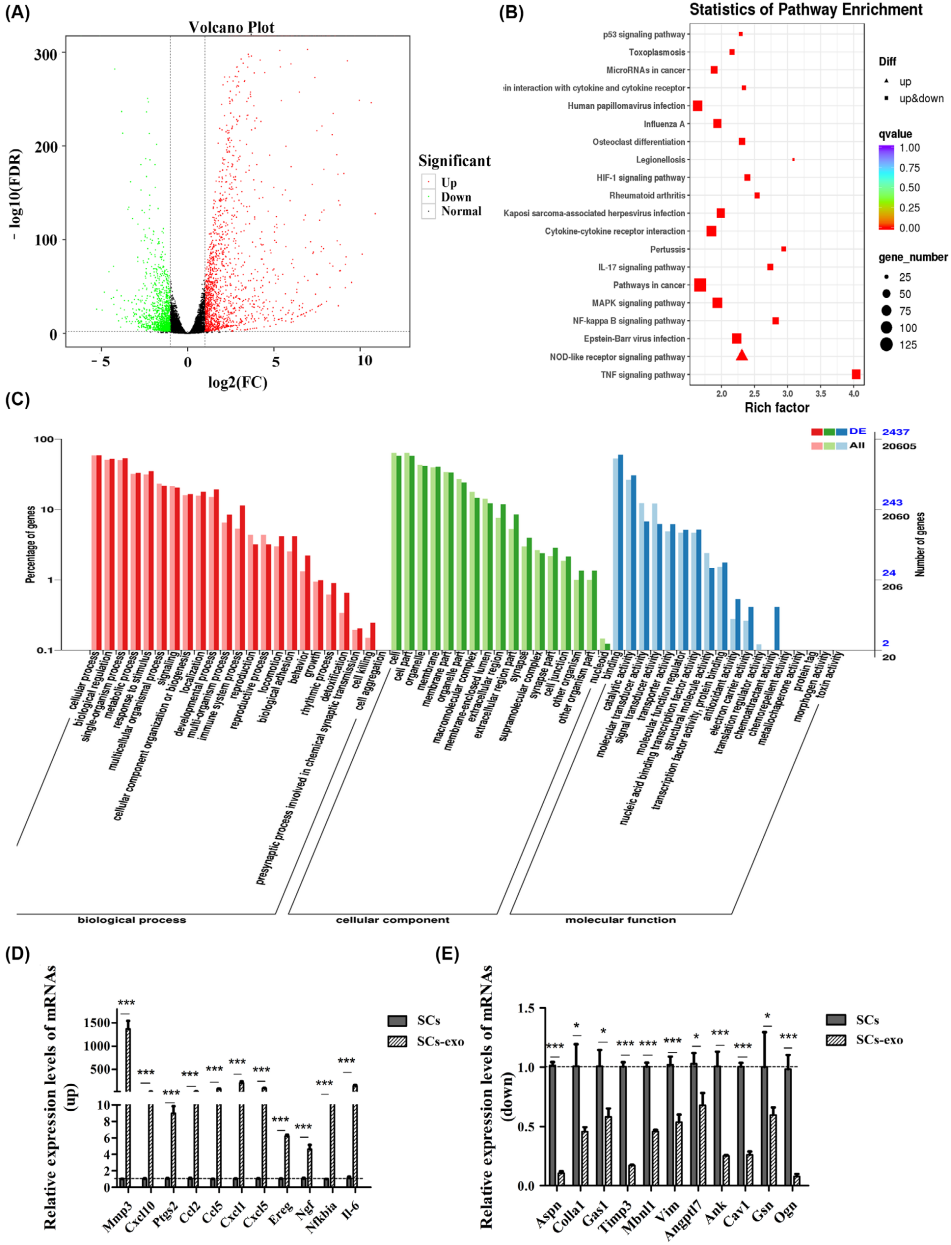
Transcriptome sequencing analysis of SCs after treatment with C2C12‐derived exosomes. (A) Volcanic map of differentially expressed genes in SCs after C2C12‐derived exosome treatment, of which 1590 genes were upregulated and 1182 were downregulated. (B) Annotation of the KEGG pathway of differentially expressed genes from SCs after C2C12‐derived exosome treatment. It is important that p53, HIF‐1, NF‐κB, IL‐17, MAPK and TNF pathways are involved in the repair process of injured muscle. (C) The annotation of GO functions of differential expression genes from SCs after C2C12‐derived exosome treatment; its main functions involve cell development process, cell metabolism, binding, etc. (D, E) Quantitative PCR verified 11 upregulated genes and 11 downregulated genes in SCs differentially expressed genes after C2C12‐derived exosome treatment (*n* = 3 per group). The error bars indicate the SEM, whereas comparisons between two groups were performed by an unpaired Student's test, *P < 0.05;***P < 0.001; SCs‐exo, C2C12‐derived exosomes processing SCs.

**Fig. 6:**
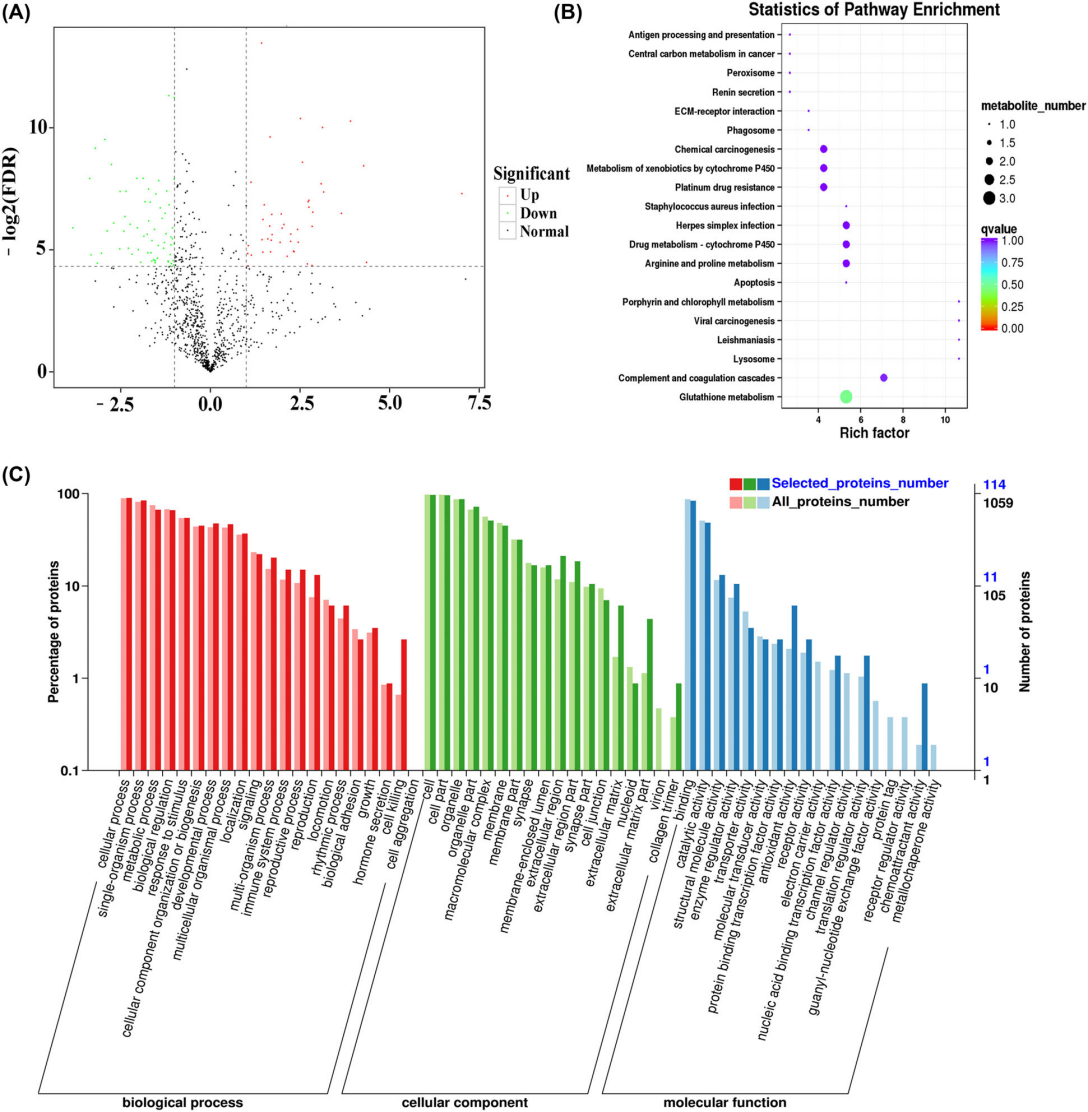
Proteomic sequencing analysis of SCs after treatment with C2C12‐derived exosomes. (A) Volcano map of SCs differential expressions of protein after C2C12‐derived exosome treatment, with 48 upregulated proteins and 66 downregulated proteins. (B) Annotation of the KEGG pathway of differentially expressed proteins, mainly focused on glutathione metabolism, complement and coagulation cascades, arginine and proline metabolism, drug metabolism‐cytochrome P450, and herpes simplex infection, which are all involved in the muscle damage repair process in different ways. (C) The classification chart of GO functions of differential expression proteins from SCs after C2C12‐derived exosome treatment.

The transcriptome sequencing detected 2772 differential genes, of which 1590 were upregulated and 1182 were downregulated (Fig. 5A). The proteomic sequencing detected 114 differential proteins, of which 48 were upregulated and 66 were downregulated (Fig. 6A). The Gene Ontology (GO; http://geneontology.org) functions mainly include cell process, cell, combination, etc. (Figs 5C and 6C).

The enrichment analysis of the Kyoto Encyclopedia of Genes and Genomes (KEGG) (https://www.genome.jp/kegg) pathways of differentially expressed genes yielded 20 pathways with the highest significance (Fig. 5B). The p53 signaling pathway mediates muscle stem cell behavior and muscle atrophy; the HIF‐1 signaling pathway is involved in postnatal muscle regeneration; the nuclear factor kappa B (NF‐κB) signaling pathway takes part in skeletal muscle atrophy mechanism; the interleukin (IL)‐17 signaling pathway participates in inflammatory response after muscle injury; the mitogen‐activated protein kinase (MAPK) signaling pathway directly engages the proliferation and differentiation of SCs; and the tumor necrosis factor (TNF) signaling pathway regulates skeletal muscle cell differentiation and the apoptosis of FAPs. Muscle‐related mRNAs (│log_2_FC│ ≥ 2.0) (Table S1) enriched in these pathways were screened, for which relative expression levels were consistent with the transcriptome sequencing results (Fig. 5D,E).

The top 20 KEGG pathways of differentially expressed proteins with the smallest *q*‐value were selected for display (Fig. 6B). There were five pathways with significance: the glutathione metabolism plays a part in protein glycosylation modifications in skeletal muscle; the complement and coagulation cascades are involved in muscle injury; the arginine and proline metabolism participates metabolism in muscle diseases; the drug metabolism‐cytochrome P450 takes part in the inflammatory signaling cascades; and the herpes simplex infection engages immunoreaction. Eight upregulated proteins and six downregulated proteins associated with muscle development were further screened (Table S2).

## Discussion

When damaged, skeletal muscles initiates a repair and regeneration program. Muscle cells can secrete nanoparticles with exosome properties to affect the proliferation and differentiation of recipient cells [19, 20]. Adipose stem cell‐derived extracellular vesicles can prevent muscle damage and inflammation in mouse models of hindlimb ischemia [21]. Therefore, we extracted C2C12‐derived exosomes, and the identification results were in agreement with the results of Linton *et al*. [12]. Exosomes were injected into the GAS of BaCl_2_‐injured mice. Combining our previous research with HE and EB staining, we knew that the damage on day 1 was not obvious, whereas the damage on day 3 was severe; the repair began on day 5, and the repair and intact muscle fiber appeared on day 7; the muscle fiber regeneration was basically completed from day 9. The exosome therapy effect of this trial was remarkable on day 5. Therefore, the present study focused on the expression changes in muscle repair and regeneration‐related genes of Pax7, MyoD, fiber‐related genes collagen‐1, α‐SMA, and fat‐related gene PPARγ in the two stages of days 3 and 5. The results showed C2C12‐derived exosomes can promote the regeneration of skeletal muscle, accelerate the production of fat from damaged muscles, and inhibit the fibrosis of damaged muscles, thereby accelerating injury repair.

SCs are of great significance for the growth and development of skeletal muscle repair and regeneration, which is located between the substrate and the muscle membrane [6], and participate in the regeneration and functional recovery of skeletal muscle, comprising the engine of muscle repair [22, 23, 24]. In skeletal muscle, SCs are usually at rest and are activated in response to muscle damage [25, 26]. After SCs are activated, they enter the cell cycle and engage the differentiation and fusion of muscle fibers to repair the damaged area. In this experiment, exosomes were acted on SCs, which significantly increased the PCNA protein expression and proliferative activity of SCs, whereas the Myod protein expression decreased. After skeletal muscle‐derived exosome treating SCs, Myod expressions decreased, indicating that skeletal muscle exosomes exhibit inhibitory effects on the differentiation of SCs [19]. The PCNA protein was involved in DNA replication and highly expressed in the S phase of the cell cycle. The PCNA was expressed in the activated SCs of mice, promoting wound healing [27]. Electro‐acupuncture promoted skeletal satellite cell proliferation by upregulating PCNA expression [28]. Our findings coincide with the above reports.

After skeletal muscle is damaged, FAPs dynamically produce primary cilia, which play a key role in regulating FAPs adipogenesis by controlling the activity of the Hedgehog signaling pathway. MMP‐14 inhibits C/EBPδ and PPARγ in FAPs through the Hedgehog signaling pathway, thereby reducing the lipogenesis of FAPs [29]. These data suggest that, in models of acute muscle injury and muscular dystrophy, the ability of FAPs to differentiate into adipocytes is reduced. Moreover, after being given high‐fat feed, mice with muscular dystrophy have a positive effect with respect to improving their phenotype, restoring mitochondrial function in FAPs, and reconnecting key signaling networks and protein complexes [30]. We applied C2C12‐derived exosomes to FAPs and found that they promoted the differentiation of FAPs into fat cells and inhibited the conversion of FAPs to fibrosis, providing new ideas for improving muscle damage and muscular dystrophy. However, the mechanism of action needs to be investigated further.

Currently, the amount of research on exosomes mediating cell‐to‐cell communication under physiological and pathological conditions is increasing. In particular, skeletal muscle exosomes play a fundamental role in the dynamic balance and development [31, 32]. Skeletal muscle cells release protein/nucleic acid complexes in microvesicles, promoting myogenesis and muscle regeneration [33, 34, 35]. Myotube‐derived exosomes can promote myoblast differentiation by altering the expression of cyclinD1 and myogenin [36]. To explore the mechanism by which C2C12‐derived exosomes regulate SCs and thus promote repair and regeneration, we performed transcriptome and proteome differential expression analysis of C2C12‐derived exosome‐treated SCs (SCs‐exo) and screened for signaling pathways and important genetic material that may play a major role in damage repair. In the transcriptome data, p53 has a role in mediating muscle stem cell activation and muscle atrophy, indicating new targets for treating muscle atrophy [37]. HIF1α and HIF2α are necessary to maintain SCs self‐renewal in an oxygen‐deficient environment [38]. The activation of NF‐κB is key to mediating the entire process of muscle atrophy [39]. Gga‐miR‐3525 Targets PDLIM3 through the MAPK signaling pathway to regulate the proliferation and differentiation of skeletal muscle SCs [40]. TNF‐α modifies microRNA signatures in skeletal muscle cell differentiation [41]. Nilotinib reduces muscle fibrosis in chronic muscle injury by promoting the TNF‐mediated apoptosis of FAPs [42]. MMP3 is the most significant upregulated gene in the differentially expressed genes. ASC‐exosomes inhibit scar formation by preventing fibroblast fibrosis and improving the ratio of COLI/III, TGFβ3/TGFβ1, MMP3/TIMP1 [43]. In the proteomic data, glutathione depletion and acute exercise affect the glycosylation modification of proteins in skeletal muscle in mice [44]. Arginine and proline metabolism are associated with muscular dystrophy in the Golden retriever [45]. Drug metabolism‐cytochrome P450 is involved in the inflammatory response [46] and its expression is induced by high‐fat feeding and skeletal muscle IL‐6 dependence [47]. Differentially expressed proteins that may be related to myogenesis mechanisms were mined (Table S2). Tyrosine‐protein phosphatase non‐receptor type 11 acts on various receptors and downstream of the cytoplasmic protein tyrosine kinase, which involves signal transduction from the cell surface to the nucleus. Complement C3 plays a central role in the activation of the complement system, and complement‐mediated muscle damage is at the heart of dysferlinopathy pathogenesis, suggesting that targeting the complement system may be a treatment for the disease [48]. SPARC appears to be an important regulator of actin cytoskeletons, which are involved in the maintenance of muscle function [49]. Upregulation of heme oxygenase‐1 by hemin alleviates sepsis‐induced muscle wasting in mice [50]. Galectin‐3 and *N*‐acetylglucosamine promote muscle production and improve skeletal muscle function in muscular dystrophy models [51]. In summary, C2C12‐derived exosomes promote SCs proliferation by regulating muscle‐related mRNA and protein expression, thus promoting muscle repair and regeneration.

## Conflict of interest

The authors declare no conflict of interest.

## Author contributions

Most of the experiments were performed by SJ and PM; HW and SJ wrote the manuscript; JW revised the manuscript; XC performed part of the *in vitro* experiments; XY and XL performed part of the *in vivo* experiments; YY, JL, WH, and ZZ provided technical assistance; HW and YD designed the experiments and provided funds. All authors read and approved the final manuscript submitted for publication.

## Supporting information


**Table S1.** Primers sequence used in the present study.

**Table S2.** The difference in gene expression significantly between SC and SCs‐exo in the KEGG pathway (¦log_2_FC¦ > 2).

**Table S3.** Differential expression of muscle related‐proteins between SCs and SCs‐exo (¦log_2_FC¦ > 2).

## Data Availability

All data generated or analyzed during this study are included in this published article and its supplementary information files.
