# Supplementary material for: Myoblast‐derived exosomes promote the repair and regeneration of injured skeletal muscle in mice
Source: FEBS Open Bio. 2022 Nov 11;12(12):2213–26. doi: 10.1002/2211-5463.13504 (PMC9714366; doi:10.1002/2211-5463.13504)
Supplement: Supplementary file 1 — Table S1. Primers sequence used in the present study. [file FEB4-12-2213-s002.docx]

Supplementary Table 1. Primers sequence used in this study

| **Primer Name** | **Primer Sequence (5’→3’)** |
| --- | --- |
| M-PPARγ-F  M-PPARγ-R  M-MyoD-F  M-MyoD-R | AGAAGCGGTGAACCACTGATA  AGGTCCACAGAGCTGATTCC  GAATGGCTACGACACCGCCTACTAC  ACGGGGTCTGGGTTCCCTGTT |
| M-Pax7-F  M-Pax7-R  M-collagen1-F  M-collagen 1-R | TCAAGCTCCGTGTTTCTCATGG  TTGTATTCTGAGCACTCGGCT  GCAGGATCTCTATAGCATCG  TGTCTCACAGTAACTCTCCA |
| M-αSMA-F  M-αSMA-R  M-Cxcl1-F  M-Cxcl1-R  M-Ccl2-F  M-Ccl2-R | AAGAGAGGGATCCTGACGCT  AGAGGCATAGAGGGACAGCA  GGCTGGGATTCACCTCAA  GGCTATGACTTCGGTTTGG  TGGGTCCAGACATACATT  TACGGGTCAACTTCACAT |
| M-Cxcl5-F  M-Cxcl5-R | CCCTACGGTGGAAGTCATAGC  GCCCTTTCTTCTCTTCACTGG |
| M-Ccl5-F  M-Ccl5-R | CACCACTCCCTGCTGCTT  ACTTGGCGGTTCCTTCG |
| M-Ereg-F  M-Ereg-R | CTGCCTCTTGGGTCTTGACG  GCGGTACAGTTATCCTCGGATTC |
| M-Ngf-F  M-Ngf-R | CCAGTGAAATTAGGCTCCCTG  CCTTGGCAAAACCTTTATTGGG |
| M-Mmp3-F  M-Mmp3-R | ACATGGAGACTTTGTCCCTTTTG  TTGGCTGAGTGGTAGAGTCCC |
| M-Nfkbia-F  M-Nfkbia-R | TGGCAATCATCCACGAAG  CACAGGCAAGATGTAGAGGG |
| M-Cxcl10-F  M-Cxcl10-R | CCAAGTGCTGCCGTCATTTTC  GGCTCGCAGGGATGATTTCAA |
| M-Ptgs2-F  M-Ptgs2-R | TTCAACACACTCTATCACTGGC  AGAAGCGTTTGCGGTACTCAT |
| M-Aspn-F  M-Aspn-R | AAGGAGTATGTGATGCTACTGCT  ACATTGGCACCCAAATGGACA |
| M-Vim-F  M-Vim-R | AGCCTCTATTCCTCATCCC  GTTGGCAAAGCGGTCAT |
| M-Angptl7-F  M-Angptl7-R | TGACTGTTCTTCCCTGTACCA  CAAGGCCACTCTTACGTCTCT |
| M-Gsn-F  M-Gsn-R | ATGGCTCCGTACCGCTCTT  GCCTCAGACACCCGACTTT |
| M-Ogn-F  M-Ogn-R | ACCATAACGACCTGGAATCTGT  AACGAGTGTCATTAGCCTTGC |
| M-Gas1-F  M-Gas1-R | CCATCTGCGAATCGGTCAAAG  GCTCGTCGTCATATTCTTCGTC |
| M-Timp3-F  M-Timp3-R | CTTCTGCAACTCCGACATCGT  GGGGCATCTTACTGAAGCCTC |
| M-Cav1-F  M-Cav1-R | ATGTCTGGGGGCAAATACGTG  CGCGTCATACACTTGCTTCT |
| M-Mbnl1-F  M-Mbnl1-R | GCTGCCCAATACCAGGTCAA  GTAGCGGGTGTCATGCACAAT |
| M-Ank-F  M-Ank-R  M-β-actin-F  M-β-actin-R | CAGTCAAGGAGGATGCAGTAGA  CACTGTAGGCTATCAGGGTGT  TTGCTGACAGGATGCAGAAG  ACATCTGCTGGAAGGTGGAC |
