# Supplementary material for: Myoblast‐derived exosomes promote the repair and regeneration of injured skeletal muscle in mice
Source: FEBS Open Bio. 2022 Nov 11;12(12):2213–26. doi: 10.1002/2211-5463.13504 (PMC9714366; doi:10.1002/2211-5463.13504)
Supplement: Supplementary file 2 — Table S2. The difference in gene expression significantly between SC and SCs‐exo in the KEGG pathway (¦log2FC¦ > 2). [file FEB4-12-2213-s001.docx]

Supplementary Table 2. The difference in gene expression significantly between SC and SCs-exo in KEGG pathway (|log2FC|>2)

| **Pathway. Name** | **Pathway. ID** | **Gene, name** | **Regulate** |
| --- | --- | --- | --- |
| **PI3K-Akt signaling pathway** | **ko04151** | **Cxcll, Cxcl5, Ccl2, Cx3cll, Ptgs2** | **Up** |
| **MAPK signaling pathway** | **ko04010** | **Ccl5, Ereg, Ngf, Map2kl, Mapkapk2** | **Up** |
| **IL-17 signaling pathway** | **ko04657** | **Lcn2, CxcllO, Mmp3, Cxcll, Cxcl5** | **Up** |
| **NF-kappa B signaling pathway** | **ko04064** | **Cxcl2, Ddx58, Nfkbia, Lbp, Vcaml, Ptgs2** | **Up** |
| **Epstein-Barr virus infection** | **ko05169** |  | **Down** |
| **MicroRNAs in cancer** | **ko05206** |  |  |
| **Fc gamma R-mediated phagocytosis** | **ko04666** |  |  |
| **Regulation of actin cytoskeleton** | **ko04810** | **Gsn** | **Down** |
| **Viral carcinogenesis** | **ko05203** |  |  |
| **MAPK signaling pathway** | **ko04010** | **Angptl7** | **Down** |
| **__** | **—** | **Ogn** | **Down** |
| **Proteoglycans in cancer MicroRNAs in cancer** | **ko05205**  **ko05206** | **Timp3** | **Down** |
| **Endocytosis** | **ko04144** |  |  |
| **Focal adhesion** | **ko04510** |  |  |
| **Prion disease** | **ko05020** |  |  |
| **Bacterial invasion of epithelial cells Proteoglycans in** | **ko05100** | **Cavl** | **Down** |
| **cancer** | **ko05205** |  |  |
| **Viral myocarditis** | **ko05416** |  |  |
| **Fluid shear stress and atherosclerosis** | **ko05418** |  |  |
| **—** | **—** | **Ank** | **Down** |
| **--** | **--** | **Mbnll** | **Down** |
| **--** |  | **Aspn** | **Down** |
| **Hedgehog signaling pathway** | **ko04340** | **Gasl** | **Down** |
| **Protein digestion and absorption** | **ko04974** | **Collal** | **Down** |
