# Supplementary material for: Myoblast‐derived exosomes promote the repair and regeneration of injured skeletal muscle in mice
Source: FEBS Open Bio. 2022 Nov 11;12(12):2213–26. doi: 10.1002/2211-5463.13504 (PMC9714366; doi:10.1002/2211-5463.13504)
Supplement: Supplementary file 3 — Table S3. Differential expression of muscle related‐proteins between SCs and SCs‐exo (¦log2FC¦ > 2). [file FEB4-12-2213-s003.docx]

Supplementary Table 3. Differential expression of muscle related-proteins between SCs and SCs-exo (|log2FC|>2)

| **Uniprot ID** | **Protein names** | **P value** | **log2FC** | **Regulated** |
| --- | --- | --- | --- | --- |
| **P35355** | **Prostaglandin G/H synthase 2** | **0.044642154** | **4.355** | **up** |
| **P41499** | **Tyrosine-protein phosphatase non­receptor type 11** | **0.016059816** | **2.839** | **up** |
| **P01026** | **Complement C3** | **0.048809638** | **2.835** | **up** |
| **Q9JKS6** | **Protein piccolo** | **0.007970452** | **2.723** | **up** |
| **Pl6975** | **SPARC (Basement-membrane protein**  **40)** | **0.025173642** | **2.451** | **up** |
| **P05370** | **Glucose-6-phosphate 1 -dehydrogenase** | **0.017501875** | **2.431** | **up** |
| **P06762** | **Heme oxygenase 1** | **0.032992151** | **2.335** | **up** |
| **P08699** | **Galectin-3** | **0.024553178** | **2.237** | **up** |
| **Q68A21** | **Transcriptional activator protein Pur­beta** | **0.00411683** | **-3.367** | **down** |
| **Q62868** | **Rlio-associated protein kinase 2** | **0.036026267** | **-3.328** | **down** |
| **Q63610** | **Tropomyosin alpha-3 chain** | **0.001743166** | **-3.210** | **down** |
| **Q5RJR2** | **Twinfilin-1** | **0.004103882** | **-2.049** | **down** |
| **Q6IMF3** | **Keratin, type II cytoskeletal 1** | **0.031129225** | **-2.0125** | **down** |
| **Q64578** | **Sarcoplasmic/endoplasmic reticulum calcium ATPase 1 (SERCA1)** | **0.044436835** | **-1.944** | **down** |
